# Practical Notes and Formulæ

**Published:** 1887-12

**Authors:** 


### PRACTICAL NOTES AND FORMULÆ.

**Prurigo.**—Dr. White, of Indiana (*in Medical Age*), says of this disease: "I have tried a number of remedies in my practice, alkaline baths, sulphur ointment, Goulard's cerate, various lotions, etc., and have occasionally given iodide of potassium internally, but found nothing equal to a solution of corrosive sublimate. My prescription is:

R. Hydrarg. bichlorid ............................ ℈ ij.

Ammoniæ muriat ............................ ʒ j,

Aquæ puræ ............................ O ij.

M. Signe—Apply night and morning.

Put where children cannot get it.

The quantity of corrosive sublimate should be diminished in treating very young children. I never knew any bad effects from the use of the lotion. It relieves the itching, and will generally cure, if persevered with; and, furthermore, it is cleanly.

Internal remedies are seldom necessary. In domestic practice a decoction of poke-root, (phytolacca decandra) has been used as a lotion in desperate cases with good results; but is not safe, and the suffering caused by it can scarcely be described by those who have been tempted to give this remedy a trial.

**Local Anæsthetic.**—Dr. T. B. Wood asks for the best local anæsthetic for extracting teeth. The best that I know of is the following, which always acts promptly:

℞. Ol. wintergreen ............................ ʒ ij,

Chloroform ............................ ʒ j,

Sulph. ether ............................ ʒ j,

Hyd. chlor ............................ ʒ ij,

Ol. cloves ............................ ʒ iv,

Alcohol ............................ ℥ iss.

M. Sig.—Apply with cotton, pressed upon each side of the tooth.—*Dr. W. T. Coggin, in Medical Brief.*

Dr. E. van Goitdsnoven, of Atlanta, Ga., suggests the following:

℞. Cocaine ............................ grs. iv,
Oil of cloves ............................ gtts. c.

**Instantaneous Remedy for Lumbago.**—The following mixture can be employed in cases of accidental lumbago, or rheumatic pain produced by strain or muscular exertion:

℞. Collodion ............................
Tr. iodini. ............................
Aquæ ammonii ............................ aa ʒ i.

M. Sig.—Apply freely and widely over the affected parts with a camel's hair brush.—*K. C. Medical Index.*

**Ointment for Scabies—**

℞. Napthali....................................pts. xv,
Saponis virid.............................pts. l,
Adipis.........................................pts. c,
Pulv. cretæ..................................pts. x.

M. One application of the above is said to be effectual. No bath is required previously, and the skin is left in good condition. —*Medical Age.*

**For Consumption—**

℞. Compound syrup of hypophosphites..........fl ℥ iv,
Whisky.........................................fl ℥ iv,
Fowler's solution.............................fl ʒ ii.

Mix. The dose is one tablespoonful one-half hour before each meal.—*National Druggist.*

**Antiseptic Powder—**

℞. Iodoform,
Powdered cinchona,
Powdered benzoin,
Powdered carbonate of magnesium, equal parts,
Oil of eucalyptus, q. s.

Mix the powders and saturate them with the oil.—*National Druggist.*

**Solution of Antifebrin.—**

℞. Antifebrin....................................fl ʒ ss,

Alcohol.........................................fl ʒ i,

Port wine...............................q. s. ad fl ℥ ii.

Dissolve the antifebrin in alcohol with the aid of a gentle heat, and add the port wine.—*National Druggist.*

**Mixture for Flatulent Dyspepsia.—**

℞. Tinct. gentian,
Tinct. of star-anise, } each............ parts viii,
Tinct. of nux vomica,
Chloroform ..........................parts ii–iv.

From eight to ten drops to be taken before each meal, in a wine glassful of water.—N. *Y. Medical Journal.*

**A Mixture for Infantile Diarrhœa—**

℞. Fennel-water..................................parts lxxv,
Lime-water......................................parts vi,
Subnitrate of bismuth.......................parts iii,
Syrup of orange-flowers....................parts xv.

A teaspoonful to be given every two hours to infants whose dejections are green and contain masses of undigested casein. Farinaceous food should be withheld.—*N. Y. Medical Journal.*

**Sparteine,** the alkaloid of scoparius or broom, is an oily liquid substance, and has similar physiological properties to digitalis, but instead of irritating the stomach, it acts as a tonic. The sulphate is the preparation employed, and is given in doses ranging from gr. ¼–ij.

**An Anodyne for Dental Caries.**—The following mixture is recommended:

℞. Camphor ........................................ parts v,
Chloral hydrate .................................. parts i,
Cocaine hydrochloride ........................ part i.

On heating this mixture to the boiling point of water, an oily liquid is formed. This is to be applied lightly, and it is said that after a few applications the pain will surely be alleviated.—*Physician and Surgeon.*

**To Prevent Waste of Tissue.**—The following mixture I have found to be an excellent preparation in wasting disease—in tuberculosis, convalescence from pneumonia, general anæmia, scrofula, mollities ossium, etc.:

℞. Hoff's malt extract ................................ Oj,
Syr. lactophosphate lime ........................ ℥ vj,
Crystaline phosphates ............................ ℥ iij,
Syr. auranti cort .................................... ℥ iv,
Best French brandy ................................ ℥ vj.

M. et S. From one teaspoonful to a tablespoonful, according to age, every two, three or four hours. The crystalline phosphates should be rubbed up with a little of the syrup before adding to the whole amount.—*American Medical Journal.*

**Treatment of Diabetes.**—A convenient formula for the treatment of diabetes by lithium in pill form is given in the *Therapeutic Gazette*, as recommended by Vizier:

℞. Lithii carbonat ................................... gr. iss,
Sodii arseniat ....................................... gr. 1-25.
Ext. gentian ......................................... gr. ¾.

To be taken morning and night, and continued until sugar has disappeared from the urine.—*College and Clinical.*

**For Bronchitis.**—There is no combination from which I derive so much satisfaction in the treatment of ordinary "colds" as

℞. Ammonii chloridi ................................ ʒ j,
Tinct. opii camphoratæ ........................ f ℥ ss,
Syrup scillæ comp ................................ f ℥ jss.

Teaspoonful every two or three hours, as the cough may require. If there be some fever, I add a suitable quantity of tincture of aconite.—*Medical World.*

**For Pharyngitis.**—As a "gargle," I derive most benefit, in acute inflammation of the pharynx, from:

℞. Potassi chloridi ................................... ʒ j,
Aquæ destillat ....................................... ℥ iij,
Ft. solut. et adde: tint. ferri chloridi ............... f ʒ ij.

M. Sig. Use as a gargle four or five times daily. Sometimes, if the inflammation be severe and accompanied by constitutional disturbances, I prescribe internally tincture of phytolacca decandra, with happiest results.—*Medical World.*

